# Poly(glycerol itaconate) Crosslinking via the aza-Michael Reaction—A Preliminary Research

**DOI:** 10.3390/ma16237319

**Published:** 2023-11-24

**Authors:** Magdalena Miętus, Krzysztof Kolankowski, Tomasz Gołofit, Paweł Ruśkowski, Marcin Mąkosa-Szczygieł, Agnieszka Gadomska-Gajadhur

**Affiliations:** 1 Faculty of Chemistry, Warsaw University of Technology, Noakowskiego 3 Street, 00-664 Warsaw, Poland; magdalena.mietus.dokt@pw.edu.pl (M.M.); krzysztof.kolankowski.dokt@pw.edu.pl (K.K.); tomasz.golofit@pw.edu.pl (T.G.); pawel.ruskowski@pw.edu.pl (P.R.); 2 Faculty of Natural Sciences, Department of Chemistry, Norwegian University of Science and Technology, 7034 Trondheim, Norway; marcin.k.makosa-szczygiel@stud.ntnu.no

**Keywords:** unsaturated glycerol polyesters, aliphatic diamines, post-polymerisation modification, tissue engineering

## Abstract

In unsaturated glycerol polyesters, the C=C bond is present. It makes it possible to carry out post-polymerisation modification (PPM) reactions, such as aza-Michael addition. This reaction can conduct crosslinking under in-situ conditions for tissue engineering regeneration. Until now, no description of such use of aza-Michael addition has been described. This work aims to crosslink the synthesised poly(glycerol itaconate) (PGItc; P3), polyester from itaconic acid (Ac_Itc_), and glycerol (G). The PGItc syntheses were performed in three ways: without a catalyst, in the presence of *p*-toluenesulfonic acid (PTSA), and in the presence of zinc acetate (Zn(OAc)_2_). PGItc obtained with Zn(OAc)_2_ (150 °C, 4 h, G:Ac_Itc_ = 2:1) was used to carry out the aza-Michael additions. Crosslinking reactions were conducted with each of the five aliphatic diamines: 1,2-ethylenediamine (1,2-EDA; A1), 1,4-butanediamine (1,4-BDA; A2), 1,6-hexanediamine (1,6-HDA; A3), 1,8-octanediamine (1,8-ODA; A4), and 1,10-decanediamine (1,10-DDA; A5). Four ratios of the proton amine group: C=C bond were investigated. The maximum temperature and crosslinking time were measured to select the best amine for the addition product’s application. FTIR, ^1^H NMR, DSC, and TG analysis of the crosslinked products were also investigated.

## 1. Introduction

**Figure 1:**
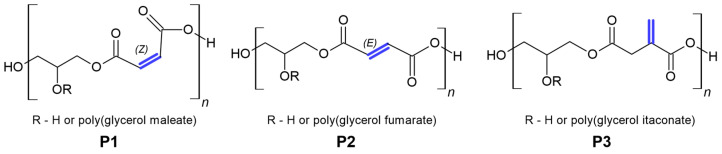
PGMal (**P1**), PGF (**P2**) and PGItc structures (**P3**).

In the structure of some glycerol polyesters, such as poly(glycerol maleate) (PGMal; P1), poly(glycerol fumarate) (PGF; P2) and poly(glycerol itaconate), an unsaturated C=C bond is present (Figure 1) [1,2,3]. It enables post-polymerisation modification [4,5,6]. Such reactions make obtaining products with different properties and structures possible [7,8,9,10].

**Figure 2:**
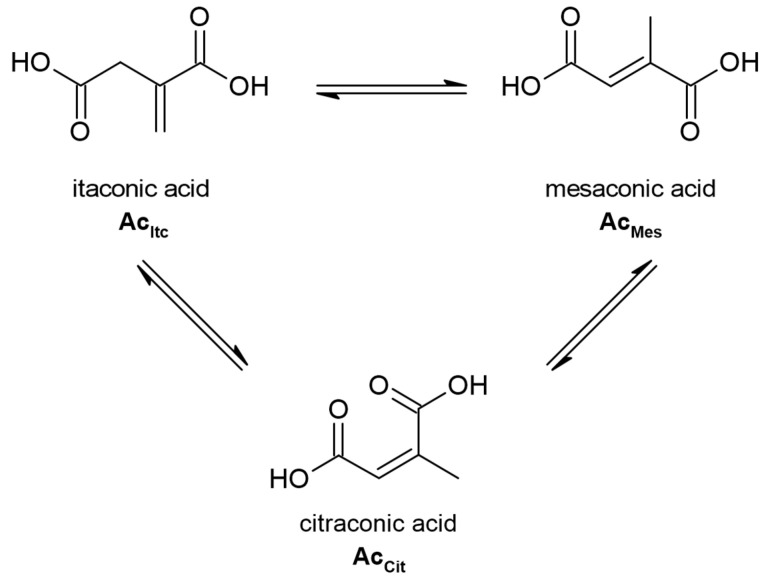
Isomerization of itaconic acid.

The investigated polymer must be soluble in organic solvents to conduct post-polymerisation reactions. The polymer should not be crosslinked or highly isomerized [6]. Itaconic compounds can isomerise to less reactive mesaconic and citraconic compounds (Figure 2) [11,12,13,14,15,16].

To minimise the isomerisation of itaconic compounds, their synthesis should be carried out at temperatures below 150 °C [11,13]. Increasing the synthesis reaction time and using a catalyst are also beneficial [11]. In the poly(glycerol itaconate) studied in this article, one of the substrates is glycerol. Due to the presence of three hydroxyl groups, there is the possibility of gelation of the resulting product [17]. It makes it impossible to carry out post-polymerisation reactions. For this reason, it is necessary to use a molar ratio of reactants other than 0.33–1.33:1 (glycerol:diacid) [17,18,19]. As with the prevention of the isomerisation reaction, it is also beneficial to lower the reaction temperature and use a catalyst or solvent [17,20,21].

The post-polymerisation reactions that unsaturated glycerol polyesters can undergo are, in particular, thermal crosslinking, photoinduced crosslinking, and Michael and Diels–Alder additions [3,7,22,23,24,25,26].

Among Michael reactions, for instance, there are aza-Michael, thio-Michael, and oxo-Michael reactions. They differ in the form of the used nucleophile (Michael donor). Considering the reactivity of the used nucleophile, the thio-Michael addition is the fastest. Michael reactions can occur at room temperature without needing a catalyst or solvent [3,7,27]. However, using a catalyst or solvent potentially makes it possible to increase the selectivity of the reaction toward Michael addition. A wide choice of substrates characterises [7,28] Michael reactions—both Michael donors and acceptors [7]. During the classic Michael reaction, a single carbon-carbon bond is formed [7]. Then, a practically irreversible reaction occurs between the α, β-unsaturated bond of the electrophile (Michael acceptor) and the nucleophile [28]. Elevated temperatures allow the Michael reaction to be partially reversed [11].

**Figure 3:**
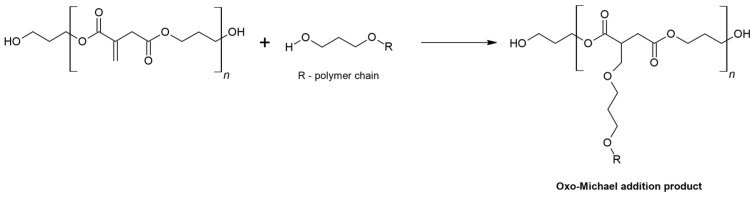
Oxo-Michael addition.

In the oxo-Michael reaction (Ordelt reaction), the nucleophile is the reactant whose structure includes a hydroxyl group [4,29]. The Ordelt reaction is one of the principal causes of the gelation of the reaction products of itaconic compounds with alcohols (Figure 3). 

**Figure 4:**
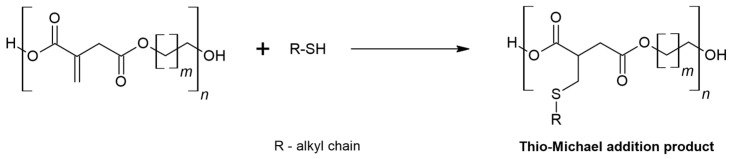
Thio-Michael addition.

To reduce the contribution of the oxo-Michael reaction, a catalyst, zinc acetate is used to synthesise itaconic compounds [20]. In the thio-Michael reaction, the thiol performs as a nucleophile (Figure 4) [30,31]. The reaction does not require solvents [5,30,32,33].

In the case of an aza-Michael addition, the role of the Michael donor may be performed by a primary or secondary aliphatic, or aromatic amine, amide, azide, or carbamate [7,30]. Michael’s acceptor may be an electron-deficient compound (with an unsaturated bond) [7,30]. It could be PGMal, PGF, or PGItc.

**Figure 5:**
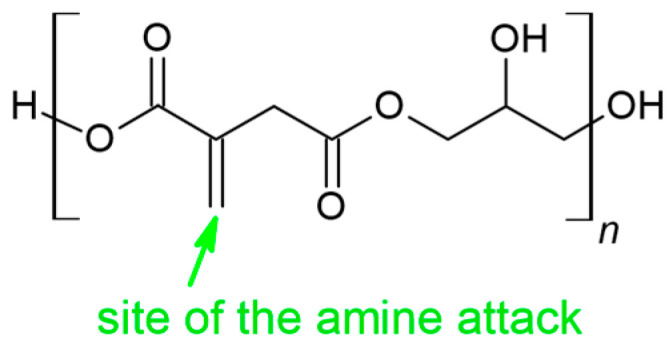
The preferred site of the amine attack in the aza-Michael addition of PGItc.

In poly(glycerol maleate) and poly(glycerol fumarate), the multiple bond occurs in the main chain of the polymer [34,35]. In the case of poly(glycerol itaconate), the multiple bond occurs in the side chain of the polymer [34]. The aza-Michael reaction of itaconic compounds occurs on the side of the carbon atom at the β position in the C=C bond (Figure 5) [36].

**Figure 6:**
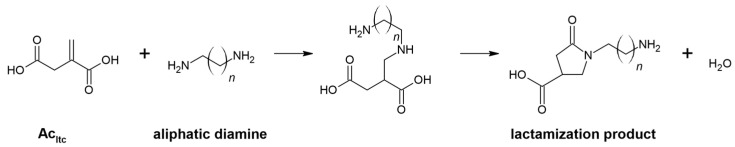
Aza-Michael addition between itaconic acid and aliphatic diamines.

It is important to consider the possibility of undesirable side reactions—lactamization (Figure 6) and isomerisation—while performing the aza-Michael addition of itaconic compounds [28,29,37].

Nevertheless, primary amines are more commonly used in aza-Michael additions than secondary amines [38,39]. It is caused by the steric hindrance of higher amines. It leads to deceleration or inhibition of the aza-Michael addition [39].

**Figure 7:**
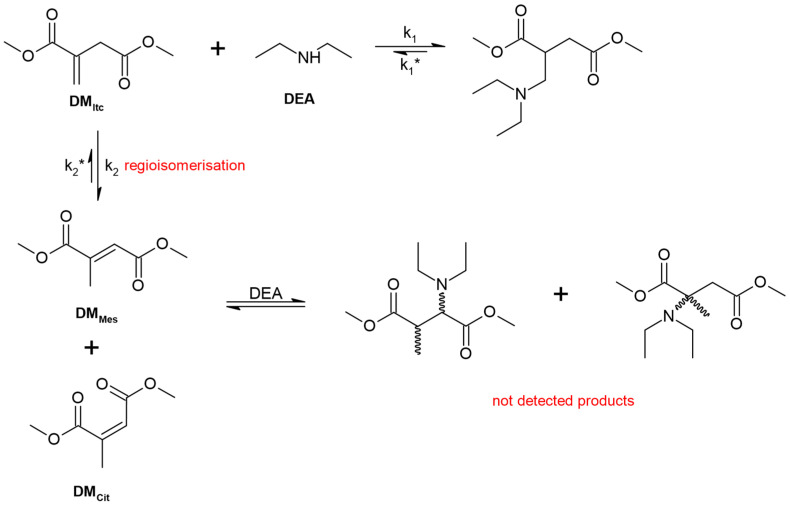
Aza-Michael addition to dimethyl itaconate using diethylamine, including isomerization (*—reverse reaction).

Amines can act as catalysts for isomerisation reactions of itaconic compounds [29,40]. Mesaconic and citraconic compounds are less reactive or unreactive towards amines than itaconic compounds [29,41]. For this reason, it may be necessary to extend the time of the aza-Michael reaction to reisomerize mesaconic and citraconic compounds to itaconic compounds [29,38]. It is also beneficial to use excessive amine to reduce the proportion of mesaconic isomers [6,42]. In 2019, a published study investigated the aza-Michael addition using dimethyl itaconate (DM_Itc_) as the Michael acceptor. The Michael donor was diethylamine (DEA) [29]. DMI underwent an isomerisation reaction to dimethyl mesaconate (DM_Mes_) and, in a small amount, to dimethyl citraconate (DM_Cit_) (Figure 7) [29].

Using a catalyst or solvent in the reaction system is favourable to reducing the proportion of side reactions occurring during aza-Michael addition [29].

In the present study, polycondensation reactions between itaconic acid and glycerol were performed to obtain poly(glycerol itaconate) [11,34]. Itaconic acid is a biocompatible, dicarboxylic chemical compound [1,2]. It is one of twelve substances considered the best value-added chemicals by the US Department of Energy [43,44]. IA has anti-cancer, antibacterial, and anti-inflammatory properties [45,46]. Dimethyl itaconate is also an interesting reactant. Like itaconic acid, it exhibits anti-inflammatory properties [47]. DM_Itc_ has better electrophilic properties than Ac_Itc_ [48]. The presence of esterified carboxyl groups increases the DMItc C=C multiple bond vulnerability to nucleophilic attack [48]. Glycerol is a biocompatible polyol with three hydroxyl groups [17,22]. It is used in the food, cosmetic, and pharmaceutical industries [49]. A notable advantage of glycerol is its FDA approval as a material for medical use [22].

**Figure 8:**
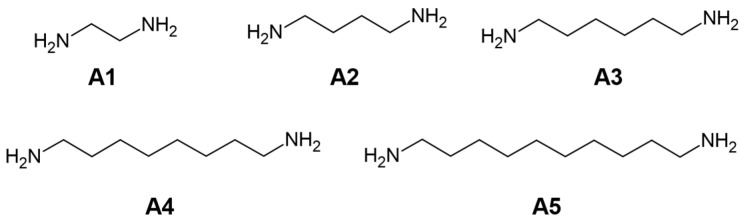
Amines studied in the experimental section.

We assume that the presented aza-Michael addition could be used as a biodegradable filler with implemented cells for direct tissue regeneration (via in-situ material injection). To confirm our hypothesis, five aliphatic diamines were tested for the aza-Michael reactions: 1,2-ethanediamine (*T*_m_ 8.3 °C, LD_50_ = 866), 1,4-butanediamine (27.7 °C, 1625), 1,6-hexanediamine (41.5 °C, 830), 1,8-octanediamine (52.0 °C, 500), and 1,10-decanediamine (62.5 °C, -) (Figure 8) [50,51].

## 2. Materials and Methods

### 2.1. NMR

To perform the NMR analysis of the samples, 130.00 to 160.00 mg of the obtained polymer or aza-Michael product was weighed into vials on an analytical balance (RADWAG, Radom, Poland). Then, 1 mL of deuterated DMSO (for polymer) (Deutero GmbH, Kastellaun, Germany) or D_2_O (for aza-Michael product) (Deutero GmbH, Kastellaun, Germany) was added. The prepared samples were closed with a cap and placed on a vibrating shaker (Heidolph 545-10000-00, Schwabach, Germany) to dissolve the vial’s contents. After this, a 700 μL sample was taken with an automatic pipette (Carl Roth GmbH + CO., Karlsruhe, Germany) and loaded into a glass tube. Then, the prepared sample underwent NMR analysis. NMR spectra were performed using a 400 MHz spectrometer (Agilent, Santa Clara, CA, USA). To analyze the NMR spectra, the program MestReNova (version 6.0.2-5457) was used.

### 2.2. FTIR

To perform IR analysis of the samples, small amounts of the polymer, aza-Michael product or standards were weighed into the vials using a technical balance (Mettler Toledo, Warsaw, Poland). IR analyses were carried out using an ALPHA spectrometer (Bruker, Berlin, Germany). The measurements used the technique of Attenuated Total Reflectance (ATR). For each sample, 32 scans in the 400–4000 cm^−1^ range were performed and averaged.

### 2.3. DSC Analysis

A Q2000 DSC analyser (TA Instruments, Eschborn, Germany) was used to perform the DSC analysis. The analysis was performed on samples weighing approximately 12 mg. The applied sample mass was determined by the sensitivity of the analysis apparatus. The procedure for DSC analysis was as follows. The first step was to cool the sample to −100 °C. The specimen was then heated to 200 °C (10 °C/min step). Next, the sample was again cooled to −100 °C. In the final stage, the sample was heated again to 200 °C. DSC thermograms were analysed using TA Instruments Universal Analysis 2000 software. Glass temperatures were determined as MidPoint temperatures of glass transitions. DSC analyses were conducted in the nitrogen flow.

The samples were heated twice. The second heating was performed because there could be an unreacted amine/polymer in the reaction system. During the first heating, the results of the aza-Michael addition reactions conducted were shown. The second heating was performed to investigate the properties of the fully reacted product.

### 2.4. TG Analysis

An SDT Q600 analyser (TA Instruments, Eschborn, Germany) was used to perform the TG analysis. The analysis was performed on samples weighing from 6 to 9 mg. The weight loss of the samples was analysed in the temperature range: room temperature—500 °C (10 °C/min step). TG thermograms were analysed using TA Instruments Universal Analysis 2000 software. TG analyses were conducted in the nitrogen flow.

### 2.5. Acid Number (AN)

A 0.5–1.0 g of the tested sample was weighed and dissolved in 25.00 mL of methanol (Chempur, Piekary Śląskie, Poland). Then, three drops of thymol blue were added. After that, the solution was titrated with a 1 M aqueous NaOH (Chempur, Piekary Śląskie, Poland) solution until the first colour change was obtained (from yellow to blue).

The acid number was calculated using the following formula:

AN [mg_KOH_/g_sample_] = ((V − V_0_) × M_NaOH_ × 56.1)/m (1)

where

V—the volume of 1 M NaOH solution used to titrate the sample [cm^3^];
V_0_—the volume of 1 M NaOH used for blank titration [cm^3^];
M_NaOH_—the titer of the solution for the titration (1 M);
56.1—the molar mass of KOH [g/mol];
m—the weight of the sample [g].

The final result is the average of three determinations.

### 2.6. Ester Number (EN)

A 0.2–0.5 g sample was weighed and dissolved in 15.00 mL of methanol and 20.00 mL of 1 M aqueous NaOH solution. The prepared solutions were refluxed for 1 h. After that, the mixture was cooled down to room temperature. Then, two drops of phenolphthalein were added. The excess NaOH was titrated with a 1 M aqueous hydrochloric acid (Chempur, Piekary Śląskie, Poland) solution until discoloured.

The ester number was calculated using the following formula:

EN [mg_KOH_/g_sample_] = (((V_0_ − V) × M_HCl_ × 56.1)/m) − AN (2)

where

V—the volume of 1 M HCl solution used to titrate the sample [cm^3^];
V_0_—the volume of 1 M HCl used for blank titration [cm^3^];
M_HCl_—the titer of the solution for the titration (1 M);
56.1—the molar mass of KOH [g/mol];
m—the weight of the sample [g].

The final result is the average of three determinations.

### 2.7. Esterification Degree by Titration (ED_tit_)

The esterification degree was calculated using the following formula:

ED = EN/(EN + AN) × 100%(3)

where:

EN—ester number;
AN—acid number.

### 2.8. PGItc Syntheses Procedure

PGItc syntheses were carried out in a Mettler Toledo MultiMax parallel reactor system (Schwerzenbach, Switzerland) in Hastelloy reactors. Glycerol (≥99%, Sigma Aldrich, Burlington, MA, USA), itaconic acid (≥99%, Sigma-Aldrich, St. Louis, MO, USA), p-toluenosulfonic acid (≥98,5%, Sigma-Aldrich, Steinheim, Germany) and anhydrous zinc acetate (≥99%, Alfa Aesar, Kandel, Germany) were used without prior preparation.

Glycerol (11.22 g, 0.122 mol; 20.51 g, 0.223 mol, 14.51 g, 0.158 mol; 6.68 g, 0.073 mol), itaconic acid (23.78 g, 0.183 mol; 14.49 g, 0.111 mol; 20.49 g, 0.158 mol; 28.32 g, 0.218 mol), and an additive catalyst were weighed into the reactor. The molar ratio of the used reactants was 2:3, 2:1, 1:1, or 1:3 (G:Ac_Itc_). The weight of the used reactants (G and Ac_Itc_) was 35.00 g. Reactors were supplied with a mechanical stirrer, temperature sensor, and Dean-Stark apparatus. In the first stage, the mixture was heated for over 10 min to T temperature. The temperature was held constant for ***t*** hours. After the reaction, the mixture was cooled down to room temperature. That phase lasted for 15 min.

### 2.9. Aza-Michael Addition Procedure

The following amines were used for aza-Michael reactions: 1,2-ethylenediamine (99%, Alfa Aesar, Karlsruhe, Germany), 1,4-butanediamine (99%, Acros Organics, Poznań, Poland), 1,6-hexanediamine (99.5 + %, Acros Organics, Geel, Belgium), 1,8-octanediamine (98%, Acros Organics, Poznań, Poland), and 1,10-decanediamine (98%, Angene Chemical, Nanjing, China).

Approximately 4.00 g of polymer was weighed into a glass vial for the crosslinking. Then, using a syringe, the entire amount needed (Table 1) of the preheated amine was added to the vial (it was heated in an oven to melt it). At this point, mixing began (a drill was used, with a bent syringe needle placed in its head—200 rpm), and crosslinking time was measured using a stopwatch. Periodically, the temperature of the mixture was checked with a glass thermometer. The crosslinking time measurement was terminated when the maximum temperature was reached.

Where the functionality:

- 1:2 (proton of the amine group:C=C bond) means that eight times less amine than PGItc was used molarly for the reaction, making ½ diamine hydrogen atom per C=C bond of PGItc;
- 1:1 (proton of the amine group:C=C bond) means that four times less amine than PGItc was used molarly for the reaction, making one diamine hydrogen atom per C=C bond of PGItc;
- 2:1 (proton of the amine group:C=C bond) means that twice as much amine as PGItc was used molarly for the reaction, making two diamine hydrogen atoms per C=C bond of PGItc;
- 4:1 (proton of the amine group:C=C bond) means that molar as much amine as PGItc was used in the reaction, making four diamine hydrogen atoms per C=C bond of PGItc; and
- 8:1 (proton of the amine group:C=C bond) means that twice as much amine as PGItc was used molarly for the reaction, so there are 8 diamine hydrogen atoms per C=C bond of PGItc.

### 2.10. PGItc End Groups Protection with Tert-Butanol (t-BuOH)

Based on the determined AN value of the PGItc product, the required amount of *t*-BuOH (≥99.5%, Sigma-Aldrich, Saint Louis, MO, USA) was calculated—7.21 g (0.0972 mol, 9.23 mL). To protect the end groups in the produced PGItc, the reaction system was cooled to 60 °C or 83 °C (t = 10 min) one hour before the scheduled end of the reaction to reduce the probability of tert-butanol evaporation (*T*_b_ 82 °C). Then, *t*-BuOH was added, and the reaction was run for another hour at 60 °C.

## 3. Results

### 3.1. PGItc Syntheses

**Figure 9:**
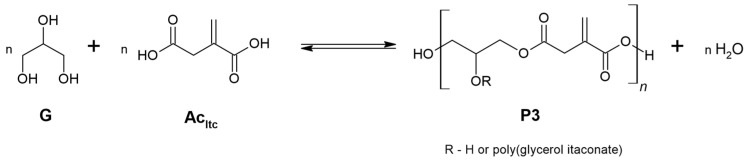
Synthesis of PGItc from glycerol and itaconic acid.

Polycondensation reactions of poly(glycerol itaconate) from itaconic acid and glycerol were carried out without the use of a catalyst and in the presence of catalysts: PTSA and Zn(OAc)_2_ (Figure 9). 

Based on titration tests, the ester number, acid number, and the degree of esterification of the obtained products were determined. Using the analyses of the ^1^H NMR spectra of the samples, the degree of esterification (ED_NMR_) and the proportion of side reactions—isomerisation to a mesaconic compound (%Is_Mes_) and Ordelt reaction (%O_rd_) were calculated. Based on the analysis of ^13^C NMR spectra, the degree of itaconic acid conversion (%X_13C_^NMR^) was calculated.

In the first stage of the study, the reactions without the catalyst were carried out (Table 2).

The samples numbered 1, 3, 9, and 10 were in the form of waxes. The remaining samples were in the form of resins, which gelled after the syntheses. Due to the lack of solubility of the samples, the 1H and 13C NMR spectra were not analysed.

Then, PGItc synthesis reactions were carried out by adding the *p*-toluenesulfonic acid catalyst (Table 3).

All the samples were in the form of wax. Due to the lack of solubility of the samples, the ^1^H and ^13^C NMR spectra were not analysed.

Finally, poly(glycerol itaconate) was synthesised with the addition of the Zn(OAc)_2_ catalyst (Table 4).

All the products of polycondensation reactions with the addition of Zn(OAc)_2_ catalyst had the consistency of the resin. 

The ^1^H NMR spectra (Figure S1) interpretation confirmed the product structure and relevant calculations.

At chemical shifts above 7.0 ppm, a signal from the protons of itaconic anhydride is visible. In the region of a chemical shift of 6.65 and 6.15 ppm, signals from protons of the isomers of itaconic acid—mesaconic acid and citraconic acid—are visible. Signals from multiple bond protons of itaconic acid are present at chemical shifts of 6.1 and 5.7 ppm. Signals from products: polyesters, oligoesters and itaconic monoesters are present at chemical shifts of 6.3–6.2 ppm and 6.0–5.75 ppm. At a chemical shift of 2.55–3.1 ppm, signals from glycerol protons are present. The value of the chemical shift depends on how the glycerol is substituted. Based on the syntheses performed, the obtained PGItc was found to have a linear structure (Figure S2). Itaconic acid has bonded to glycerol via primary hydroxyl groups. As a result, the obtained product does not undergo premature crosslinking.

The ^13^C NMR spectra (Figure S3) show signals from carbonyl carbons derived from itaconic acid, its isomers, and the products of polycondensation reactions.

**Figure 10:**
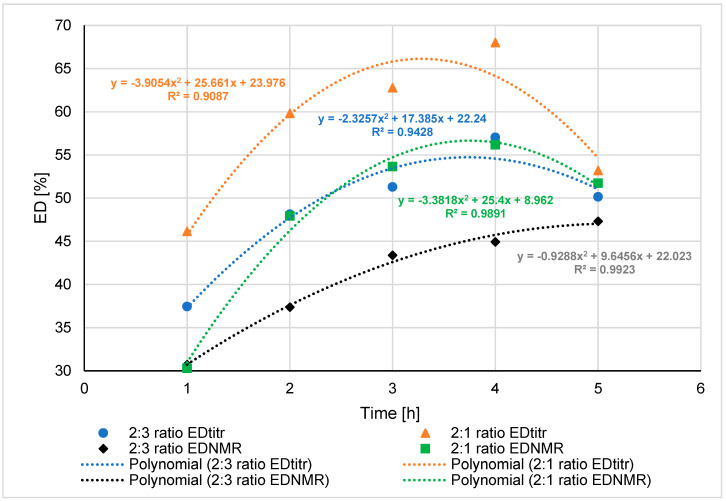
ED_titr_ and ED_NMR_ results for PGItc syntheses with Zn(OAc)_2_ catalyst.

ED_titr_ and ED_NMR_ values for the synthesised products with the Zn(OAc)_2_ catalyst were compared (Figure 10).

The degree of esterification of the synthesised products rose in reactions lasting from 1 to 4 h. For reaction products that lasted 5 h, there was a decrease in ED. It was due to the increasing amount of water in the reaction medium as the reaction progressed. The possibility of hydrolysis of ester bond in the PGItc chains was increasing. Excess glycerol increased the proportion of short PGItc chains (oligomers) in the reaction system.

For this reason, the ED for a 2:1 (G:Ac_Itc_) ratio was higher than for a 2:3 ratio. There was the highest degree of esterification at the reaction carried out at 150 °C, 4 h, and 2:1 (G:Ac_Itc_) molar ratio of substrates. The determined values of the absolute r-Pearson coefficient are close to the value of 1, which indicates a very strong correlation.

Based on the syntheses of PGItc, the crosslinking of the samples appeared to be a significant problem. It made it impossible to analyse the product by titration and NMR analysis. Many products had the resin’s consistency right after syntheses, but after a week, they took the form of gelled products. Those were obtained at temperatures above 150 °C (Table 2: reactions marked as *). For this reason, temperatures below 150 °C are required for PGItc synthesis. Higher temperatures increased the proportion of isomerisation reactions and Ordelt reactions. The temperature ≤ 120 °C was too low to obtain products that can be further analysed (Table 2, Table 3 and Table 4: reactions marked as **). 

The contribution of the isomerisation reaction of itaconic units to mesaconic units was low: 0.3–1.3% (G:Ac_Itc_ 2:3) and 0.3 1.5% (2:1). The calculated proportion of Ordelt reactions was low: 7.2–12.1% (G:Ac_Itc_ 2:3) and 7.3–14.1% (2:1).

The highest ED value was achieved when the reaction was carried out at 150 °C for 4 h. The molar ratio of the substrates was 2:1 (G:Ac_Itc_) (Table 4: reaction marked as ***). The proportion of undesired reactions was one of the highest for that product. However, it was minor, so it should not adversely affect the yield of the aza-Michael addition. The product of that reaction was tested for aza-Michael additions with five aliphatic amines.

### 3.2. Aza-Michael Reactions

**Figure 11:**
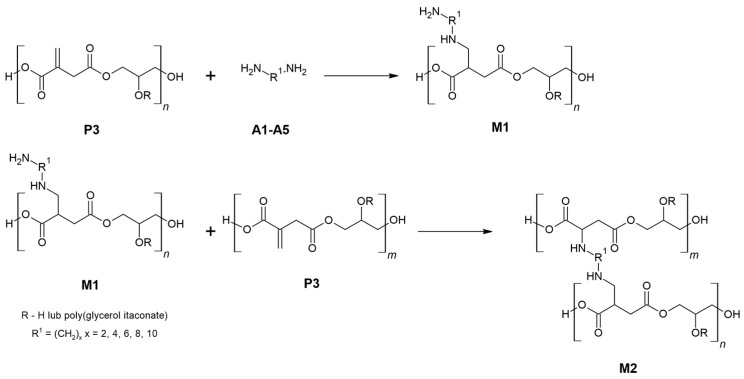
Aza-Michael addition between poly(glycerol itaconate) and aliphatic diamines.

Aza-Michael additions of five aliphatic diamines to poly(glycerol itaconate) were carried out (Figure 11). Firstly, there was a single amine attachment to the C=C multiple bonds of PGItc. Then, the crosslinking process took place. The steric hindrance in the diamine chain after its attachment to the two PGItc molecules made the formation of the tetra adduct practically impossible to observe.

For each amine, there were four crosslinking reactions. In these reactions, the ratio between the proton of the amine group: C=C bond was changed (1:2, 1:1, 2:1, 8:1). Further in the article, the ratio was called the functionality and labelled respectively: 1:2f, 1:1f, 2:1f, 8:1f.

The experiments aimed to select the best amine in terms of the previously mentioned application:

- The crosslinking product should have a dense consistency (high viscosity)—the higher the viscosity, the better the addition product is crosslinked.
- The crosslinking temperature should not exceed the temperature of 50 °C to use the addition in tissue engineering—the higher the temperature of the addition reaction, the more likely cell death is to occur.
- Crosslinking time should be as short as possible—to reduce the time of discomfort for the potential patient.

The effect of aliphatic chain length on the above parameters was also determined.

The number of repeat units in the PGItc sample (ED_titr_ = 63.7%) was calculated to determine the amount of amine required for the aza-Michael addition:

number of repeating units = 100/(100 − 63.7) = 2.76(4)

Then, the molecular weight (*M*_PGItc_) of the synthesised PGItc and the number of moles of C=C bonds in 4 g of polymer were calculated (*n*_p_):

*M*_PGItc_ = 2.76 × (number of repeating units + mass of a water molecule) = 562.28 (5)

*n*_p_ = 4/562.58 = 0.0071 mol (6)

The required numbers of moles of amines were calculated using np, followed by their masses and volumes. The aza-Michael additions were carried out without a solvent or external heat source.

#### 3.2.1. Analysis of Temperature and Time of aza-Michael Addition and the Viscosity of the Adducts

**Figure 12:**
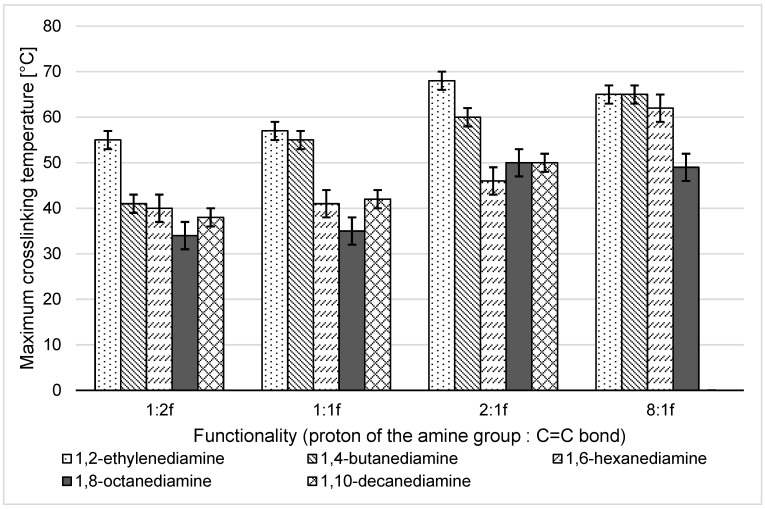
Relationship between the maximum temperature during crosslinking of PGItc and the functionality of the used substrates.

Table S1 summarises the parameters of the crosslinking reactions performed, maximum crosslinking temperatures, crosslinking durations, and visual tests of the consistency of the crosslinked products. Considering the application aspect, it was found that the samples with the best consistency were the reaction products between PGItc and 1,8-ODA (d1–d4). Their viscosity was the highest, indicating the most effective crosslinking. Due to the resulting viscosity, the least suitable amine was 1,10-DDA. The crosslinking products PGItc+1,10-DDA (e1–e4) and PGItc+1,2-EDA (a1–a4) showed the lowest viscosity, which prevented the formation of a rigid and compact structure. 1,10-decanediamine required intense heating before the reaction. In addition, the amine crystallised quickly after being scooped into a syringe. The crosslinking temperature with 1,10-DDA was very high (especially at an 8:1 ratio) (Figure 12). The large volume of the used amine contributed to forming short polymer-amine chains. The highest maximum crosslinking temperatures were observed for 1,2-EDA. Due to the very short aliphatic chain, 1,2-EDA’s mobility and ease of attachment to the C=C bond of PGItc was the highest. 

**Figure 13:**
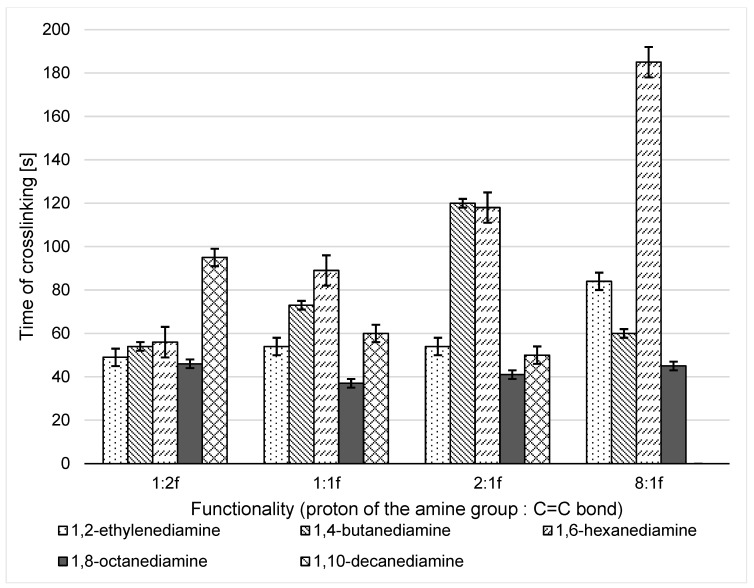
Relationship between the time of crosslinking of PGItc and the functionality of the used substrates.

Considering the maximum temperature and crosslinking time, 1,8-ODA showed the highest application potential. The crosslinking temperature of PGItc using 1,8-ODA was at a maximum of 50 °C (Figure 12) and was obtained the fastest (Figure 13).

The length of the aliphatic chains of the diamines affected the crosslinking reactions. Amines with fewer methylene groups (lower melting points), meaning 1,4-BDA and 1,6-HDA, behaved similarly to the liquid at room temperature, 1,2-EDA. In contrast, the results obtained with amines whose chains were longer (1,8-ODA and 1,10-DDA) formed the second group.

The graphs in Figure 12 and Figure 13 do not present the results for the sample with a functionality of 8:1f, because the resulting product showed unreacted, crystallised amine.

#### 3.2.2. FTIR Analysis

**Figure 14:**
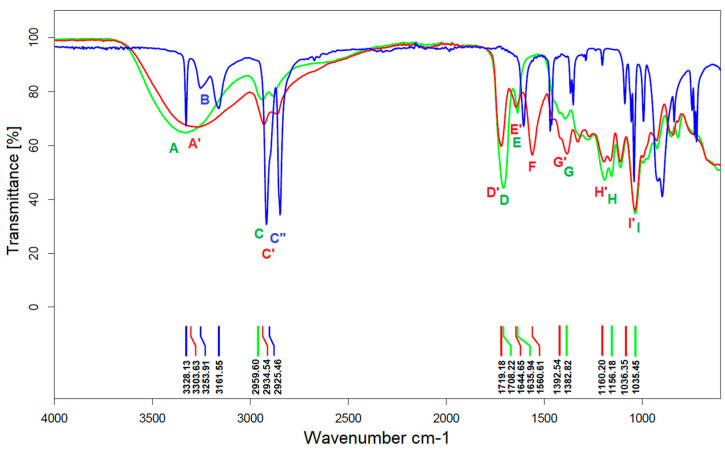
FTIR spectra of aza-Michael adduct (red), poly(glycerol itaconate) (green) and 1,8-octanediamine (blue). For detailed description of each signal see Table S2.

The structures of obtained aza-Michael products were examined by the FTIR spectrum (Figure 14). The individual signals were characterised in Table S2.

Based on the analysis of the spectra shown in Figure 14, the following conclusions were made:

- On the spectrum of the adduct, the stretching vibration of the N-H bonds of the free amine group is invisible, indicating that the amine has completely reacted with the C=C bonds of PGItc.
- The shift to larger values of the wavenumbers of D’ vibrations relative to D indicates an aza-Michael addition.
- A partial rearrangement of the C=C double bonds occurs. The weakening of the E’ vibration of the addition product compared to the E band of PGItc can be seen. However, due to the minor differences in the intensity of these vibrations, it isn’t easy to compare them reliably.
- The presence of an F vibration in the adduct indicates that an undesired reaction occurred in the reaction system between the end group (-COOH) of the polymer and the amine group of the diamine. The crosslinking products were soluble in water. In addition, the crosslinking reactions occurred without an external heat source, so there was only a small possibility for a lactamization reaction.

#### 3.2.3. NMR Analysis

NMR analyses of all crosslinked products were performed. The results of the analyses for PGItc crosslinked products with 1,8-ODA for 1:2f, 1:1f, 2:1f and 8:1f are summarised in Figures S4–S6. 

A high excess of 1,8-ODA resulted in the highest intensity signals from the amine protons (Figure S4). Similarly, the weakest signals from 1,8-ODA were observed for 1:2f. In the case of amine, the signal from A protons was not visible. It could result from the exchange of the deuterium atom from the solvent for the hydrogen atom of the amine group -NH_2_ of the amine. The signal from the amine’s A protons (protons of -NH_2_ group) was strongest for the adduct with the smallest amount of used amine. A single attachment of one amine group of the diamine molecule to one C=C multiple bonds of PGItc may have caused this. In the case of the crosslinked 1:1f sample, the signal from the A protons of the amine was invisible. That was due to the attachment of one amine group of a diamine molecule to two PGItc molecules by a C=C double bond. The lack of signals from the A protons of the amine in the 8:1f sample was due to a large amount of amine in the system—the signal from the A protons overlapped with that from the nearby protons.

No significant changes were observed in the 3.3–5.7 ppm region (Figure S5) corresponding to the -CH_2_ protons of the itaconic units and the glycerol protons in PGItc.

The most significant signals were those from multiple bond protons in itaconic acid (Ac_Itc1_, Ac_Itc2_), citraconic acid (Ac_Cit_), mesaconic acid (Ac_Mes_) and itaconic anhydride (An_Itc_). There were also signals from the protons in the C=C bond range of polyesters, oligoesters, and itaconic monoesters (Figure S6). The most significant reduction in the signals from C=C bond protons occurred in sample 8:1f. It demonstrates that the most effective attachment of the amine to the PGItc multiple bonds was with the excessive use of the amine. Using a smaller excess of amine (2:1f) also resulted in an intensity reduction of the signals from multiple bonds. In all crosslinked samples, the signal was disappearing from the C=C bond protons of itaconic anhydride.

#### 3.2.4. Differential Scanning Calorimetry Analysis

DSC analyses of PGItc, amines, and crosslinked products with solid diamines at rt (1,4-BDA, 1,6-HDA, 1,8-ODA, 1,10-DDA) for 1:2f, 1:1f and 2:1f functionalities were performed.

**Figure 15:**
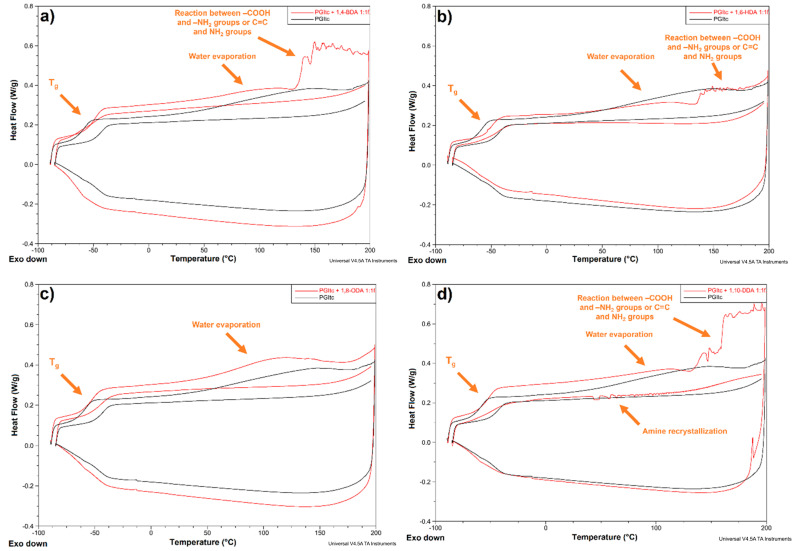
DSC analysis of PGItc sample (black), and addition products (red): (**a**) 1,4-BDA+PGItc, (**b**) 1,6-HDA+PGItc, (**c**) 1,8-ODA+PGItc, (**d**) 1,10-DDA+PGItc.

Four thermograms of the crosslinked products with 1,4-BDA (Figure 15a), 1,6-HDA (Figure 15b), 1,8-ODA (Figure 15c) and 1,10-DDA (Figure 15d) are shown for a 1:1f reactant functionality.

The first heating curves show an inflection at around −45 °C. These correspond to the glass transition process. Once the temperature exceeds 100 °C, water evaporation occurs. In the case of the crosslinked products with 1,4-BDA, 1,6-HDA and 1,10-DDA, peaks are visible after exceeding the temperature of about 125 °C. These could have two sources of occurrence. It could be a reaction between the PGItc end group and the -NH_2_ diamine groups. It results in the formation of amides. It is also possible that there is a reaction between the unreacted amine groups of diamine and the C=C bonds of PGItc. The interesting observation is that only the thermogram obtained for the PGItc+1,8-ODA product resembles the thermogram of the polymer before crosslinking. 

The presence of an inflection derived from the glass transition process and the absence of a thermal effect corresponding to the crystallisation process indicate that PGItc is amorphous.

Table S2 summarises the values of glass transition temperatures in the first and second heating cycles. The highest difference in the glass transition temperature ΔT_g_ is observed for the polymer before crosslinking.

#### 3.2.5. Thermogravimetric Analysis

**Figure 16:**
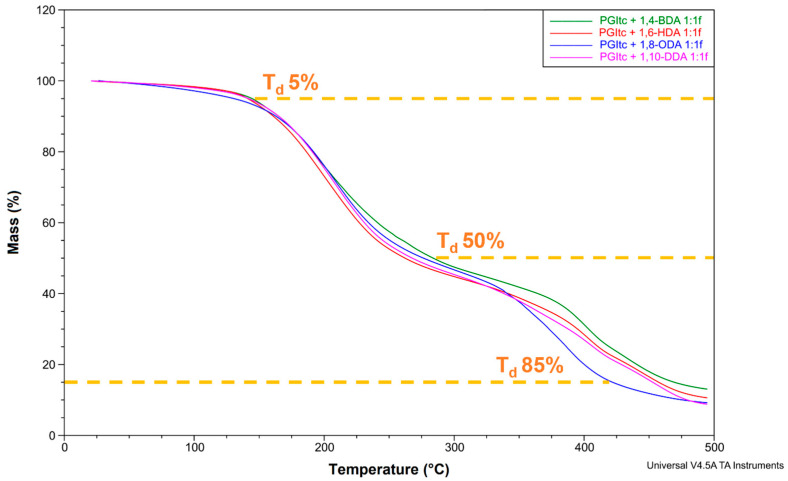
TG analysis of crosslinked samples.

TG analyses of the PGItc with solid amines were performed for a 1:1f functionality (Figure 16).

Each of the obtained TG curves has two inflection points (two-stage mass loss). Once the temperature exceeded 100 °C, absorbed water began to evaporate from the reaction system. The highest rate of this process was achieved at about 200 °C. At about 275 °C, the products were almost free of water. They were stable from about 275 °C to 325 °C. In the temperature range from 325 °C to 475 °C, the second mass loss occurred—the crosslinked product decomposition occurred. Such high decomposition temperatures conclude that the tested products show high thermal stability.

In the 350–500 °C range, the waviness are visible for the products with 1,4-BDA, 1,6-HDA and 1,10-DDA. It might indicate additional decomposition of the unreacted amine. The thermogram of the 1,8-ODA crosslinked product shows no such deviations. It confirms that the total amount of the used 1,8-ODA has reacted with PGItc.

Table S3 summarises the degradation temperatures of the crosslinked samples. The length of the used amine affects the samples’ degradation temperature (T_d_). The highest degradation temperature can be seen for the 1,4-BDA crosslinked product. As the carbon chain length of the amine increases, a tendency for Td to decrease is apparent. The exception is the product crosslinked with 1,8-ODA, which has the lowest degradation temperature.

### 3.3. PGItc End-Group Protection with Tert-Butanol

**Figure 17:**
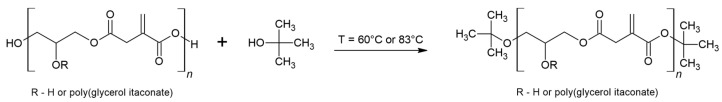
Poly(glycerol itaconate) end-group protection reaction with *t*-BuOH.

Each tested diamine used to carry out the aza-Michael addition might be involved in a side reaction—the reaction of the -NH_2_ groups of the diamine with the -COOH group of PGItc. As a result, a salt of the primary amine is formed (Figure S7). To prevent salt formation, tert-butanol (*t*-BuOH) was added during PGItc syntheses (Figure 17). The function of *t*-BuOH was to protect the polymer end groups and direct the amine to react with the multiple bonds present in PGItc.

The calculations performed to determine the required amount of *t*-BuOH (m*_t_*_-BuOH_) are shown in Supplementary Materials.

**Figure 18:**
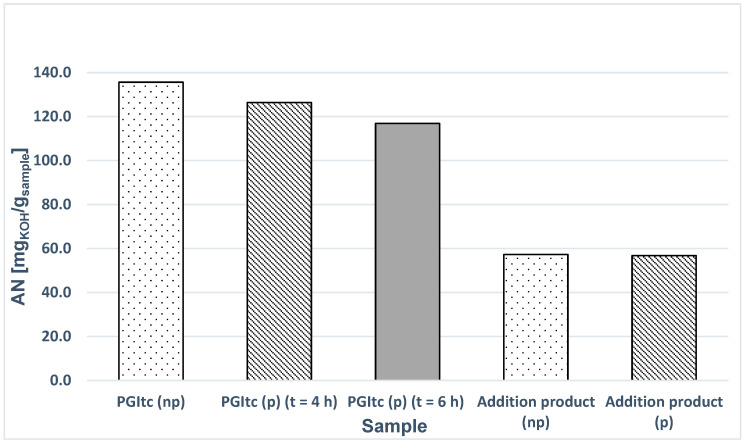
AN comparison of PGItc and crosslinking product samples.

The influence of the time of PGItc synthesis and the temperature of the reaction system on the efficiency of protecting -COOH groups in PGItc was studied. The values of acid numbers (AN) of polymers with unprotected (np) and protected (p) end groups were compared. The ANs of the unprotected and protected crosslinked products for 1:1f were also determined (Figure 18).

Based on the experiments, the following conclusions were made:

- Attempts to secure the PGItc end groups were unsuccessful. The differences between the AN values of the unprotected and protected samples are insignificant. It indicates the lack of reaction between the PGItc end group and *t*-BuOH.
- The increase in the synthesis time of PGItc has little effect on the obtained AN value.
- Performing the crosslinking reaction of protected PGItc with 1,8-ODA does not result in significant changes in the AN value compared to the crosslinked unprotected sample.

## 4. Discussion

In this work, we performed one of the first reactions to obtain poly(glycerol itaconate) as a stand-alone product. There are no articles available in the literature that are directly related to PGItc. It is generally an additive to other materials [52,53]. There was only one article where poly(glycerol itaconate) was synthesised in a reaction between itaconic anhydride and glycerol [34]. Here, we successfully performed the reaction between itaconic acid and glycerol.

The obtained poly(glyceryl itaconate) was characterised by ^1^H and ^13^C NMR analyses, according to articles by Y. Shou et al. and A. Pellis et al. reported the presence of less reactive isomers of itaconic acid—mesaconic acid and citraconic acid—in the reaction systems [11,12]. As the temperature of the polycondensation reaction was raised, the contribution of isomerisation reactions to mesaconic compounds increased. The contribution of isomerisation to citraconic compounds was negligible. According to the article by T. J. Farmer et al., this confirms the lower reactivity of citraconic isomers relative to mesaconic isomers [6]. The reactions without a catalyst and in the presence of the PTSA catalyst led to an insoluble product—generally a gel. The occurrence of an undesirable Ordelt reaction caused it [20]. According to the article by I. Schoon et al., polycondensation reactions were carried out in the presence of a Zn(OAc)_2_ catalyst [20]. It reduced the contribution of the Ordelt reaction and resulted in a resin product [20].

The literature lacks articles describing the aza-Michael addition to macromolecular itaconic compounds. Only the reactions of small-molecule compounds—itaconic acid and dimethyl itaconate—are described [28,29,37,38]. 

Despite the risk of lactamization reactions described in the article by R. Ouhichi et al., we conducted aza-Michael reactions using five aliphatic diamines [28]. The reactions were carried out without any solvent or catalyst, similar to the articles by R. Ouhchi et al., O. B. Moore et al. and D. M. Day et al. [28,29,38]. In the received addition products, there were no signals from the hydrogen atoms of the lactam ring on the ^1^H NMR spectrum. It was due to the choice of parameters for the reactions performed. The lactamization reaction requires the use of elevated temperature. In the previously mentioned article, aza-Michael additions were carried out at 50 °C [28]. 

According to R. Ouhichi et al., using amine could lead to the isomerisation of the itaconic compounds [28]. Increasing the addition time has helped reduce the proportion of isomerisation reactions [28,29,38]. The same effect could be obtained using an amine excess [29]. In the article by O. B. Moore et al. and in the aza-Michael reactions that we performed, the use of an amine in excess led to a reduction in the signals from the protons of the mesaconic and citraconic isomers on the ^1^H NMR [29]. The aza-Michael reactions performed were significantly shorter than previously reported in the literature. The obtained aza-Michael addition products using 1,8-ODA were characterised as the best viscosity, thermal and ^1^H NMR analyses. It indicates that using an amine excess while reducing the addition time has a favourable effect on the efficiency of the aza-Michael reaction.

Given the structure of the used amines, using an amine with higher steric crowding reduces the efficiency of the aza-Michael addition [38]. In the experiments conducted, it was observed that 1,10-decanediamine results in an ineffective addition. However, amines with low steric crowding could likewise result in inefficient addition. Such results were observed for reactions with 1,2-ethanediamine.

The aza-Michael reactions performed were carried out without solvents or catalysts. The article by D. M. Day et al. points out the beneficial effect of their use on the yield of the Michael adducts [38]. Therefore, in the future, it would be interesting to test the effect of their use on the efficiency of the aza-Michael addition to itaconic compounds.

Considering the possible use of the aza-Michael addition in tissue engineering, attention should be paid to a reaction’s temperature. It can be carried out at room temperature [3,7,29,38]. It is extremely significant considering that temperatures above 50 °C can be unfavourable for the growth and proliferation of cells implanted on the scaffold formed from the addition product [54,55]. However, in the literature, no attention has been given to studying parameters such as the maximum crosslinking temperature or time of the aza-Michael addition reaction. Currently used bone cements are based on acrylic compounds [56]. During the synthesis of bone cement from poly(methyl methacrylate) (PMMA), the reaction temperature could exceed 100 °C, and the curing time could last for a minimum of one hour [56,57]. Furthermore, PMMA is not absorbed in the human body, which could complicate the regeneration of damaged bone tissue [57]. Calcium phosphate-based cements (CPCs) have a shorter curing time (<10 min) [58,59,60]. However, CPCs tend to disintegrate quickly in contact with human fluids [58]. It allows us to conclude that the presented method of obtaining a bone filler from PGItc-amine adduct has a real potential to replace previous methods.

## 5. Conclusions

According to literature reports, the Zn(OAc)_2_ catalyst had a favourable effect on the solubility of the polycondensation product. Reducing the proportion of unwanted side reactions resulted in no gelation of the product. Using PTSA as a catalyst did not allow for obtaining a soluble product, which can then be crosslinked.

The performed crosslinking reactions made us conclude that PGItc is a suitable reactant for performing aza-Michael additions. Based on the reactions performed, it was found that 1,8-ODA showed the highest potential. Crosslinked products with 1,8-ODA had the most compact consistency. Crosslinking time with 1,8-ODA was shorter compared to the other amines. The crosslinking temperature for 1,8-ODA did not exceed 50 °C. This temperature should not lead to the death of cells that would be implemented on the PGItc+amine scaffold. DSC and TG analyses confirmed that the length and large rotation capacity of 1,8-ODA allow the crosslinking process to be carried out efficiently. Even though 1,10-DDA is a longer amine than 1,8-ODA, it did not allow us to obtain products with adequate characteristics. Furthermore, the crosslinking temperature using a significant excess of 1,10-DDA was high (˃80 °C). Analyses of the ^1^H NMR spectra of the crosslinked products confirmed the highest potential of 1,8-ODA.

An excess of diamine can lead to an incomplete reaction of the used amine. It can cause damage to the body’s vessels—the reaction between amines and fatty acids could occur. That reduces their barrier properties. In the case of PGItc, an excess of unreacted polymer will likely result in an oxo-Michael reaction or a radical polymerisation reaction of PGItc through the C=C bond. These reactions are not expected to affect the human body negatively. FTIR analysis found that during PGItc crosslinking, an undesirable side reaction between the amine groups of diamine and the -COOH end group of PGItc is possible. It leads to the formation of salts. The attempts made to protect the end groups of PGItc were unsuccessful. 

An interesting direction would be to perform an aza-Michael addition using poly(glycerol itaconate) derived from itaconic acid derivatives with protected -COOH groups, for instance, dimethyl itaconate. It should help prevent the salt formation reaction.

To confirm whether the aza-Michael potential bone fillers produced can be used in tissue engineering, further cell line studies should be performed in the near future.

## Figures and Tables

**Table 1:** Summary of the amounts of amines used in the PGItc crosslinking reactions.

Signature	Amine	Amine Weight [g]	Amine Volume [mL]	Functionality (Proton of the Amine Group: C=C Bond)
A1	1,2-EDA	0.05	0.06	1:2
A2		0.11	0.12	1:1
A3		0.21	0.24	2:1
A4		0.85	0.95	8:1
B1	1,4-BDA	0.08	0.08	1:2
B2		0.16	0.18	1:1
B3		0.31	0.36	2:1
B4		1.25	1.43	8:1
C1	1,6-HDA	0.10	0.11	1:2
C2		0.21	0.22	1:1
C3		0.41	0.44	2:1
C4		1.65	1.77	8:1
D1	1,8-ODA	0.13	0.13	1:2
D2		0.26	0.26	1:1
D3		0.51	0.52	2:1
-		1.02	1.04	4:1
D4		2.05	2.09	8:1
E1	1,10-DDA	0.15	0.18	1:2
E2		0.31	0.36	1:1
E3		0.61	0.71	2:1
E4		2.45	2.85	8:1

**Table 2:** Synthesis conditions and determined AN, EN, and EDtit of PGItc products in the reactions with no use of the catalyst (* reactions conducted at a temperature ˃150 °C, ** reactions conducted at a temperature ≤ 120 °C).

No.	Molar Ratio (G:Ac_Itc_)	Temperature (T) [°C]	Time (t) [min]	AN [mg_KOH_/g_sample_]	EN [mg_KOH_/g_sample_]	ED_titr_ [%]
1 **	2:3	120	60	79	442	15.2
2 **			180	n. s.	n. s.	-
3		150	60			
4			120			
5 *		165	20	385	176	31.4
6 *			45	338	245	42.0
7 *			60	315	197	38.5
8 *			120	n. s.	n. s.	-
9 *	1:3		20	526	135	20.5
10 *			45	374	187	33.3
11 *	1:1		20	308	187	37.7
12 *			45	265	241	47.7
13 *	2:3	180	30	310	294	48.7
14 *			45	298	n. s.	-
15 *			60	n. s.		
16 *			120			
17 *	1:3		45	441	529	54.5
18 *			60	n. s.	n. s.	-
19 *	1:1		45	222		
20 *			60	n. s.		

Where n. s. means not soluble.

**Table 3:** Synthesis conditions and determined AN and EN of PGItc products in the reactions with a PTSA as catalyst (** reactions conducted in the temperature ≤ 120 °C).

No.	Molar Ratio (G:Ac_Itc_)	Temperature [°C]	Time [min]	Amount of Catalyst [%_wa._]	AN [mg_KOH_/g_sample_]	EN [mg_KOH_/g_sample_]	ED_titr_ [%]
1 **	2:3	100	30	0.1	n. s.	n. s.	-
2 **		120	20	2.0			
3 **			30	0.25			
4 **				0.1			
5 **				0.5			
6 **				1.0			
7 **				2.0			
8		135		0.25			
9		150		0.05			
10				0.25			
11				0.5			
12				1.0			
13			45	1.0			
14			60	1.0			
15				2.0			

Where n. s. means not soluble. Where %_wa._ means the weight relative to the mass of the used acid.

**Table 4:** Syntheses conditions and determined AN, EN, ED_titr_, ED_NMR_, %Iz_Mes_, %O_rd_, %X_13C_^NMR^ of PGItc products in the reactions using the Zn(OAc)_2_ catalyst (** reactions conducted in the temperature ≤ 120 °C, *** reaction chosen for PGItc synthesis used for aza-Michael addition).

No.	Molar Ratio (G:Ac_Itc_)	Temperature [°C]	Time (t) [h]	AN [mg_KOH_/g_sample_]	EN [mg_KOH_/g_sample_]	ED_titr_ [%]	ED_NMR_ [%]	%Iz_Mes_ [%]	%O_rd_ [%]	%X_13C_^NMR^ [%]
1 **	2:3	120	2	406	n. s.	-	22.5	0.3	7.2	-
2		150	1	365	219	37.5	30.7	0.7	7.2	70.4
3			2	333	309	48.1	37.4	0.8	9.8	70.8
4			3	315	332	51.3	43.4	1.0	8.2	77.4
5			4	301	400	57.0	44.9	1.1	9.2	76.4
6			5	281	282	50.2	47,3	1,3	12.1	77.0
7 **	2:1	120	2	111	n. s.	-	22.5	0.3	7.3	-
8		150	1	157	135	46.2	30.3	0.7	9.9	77.8
9			2	135	201	59.8	47.9	0.9	10.8	89.4
10			3	116	195	62.8	53.7	1.3	11.1	92.6
11 ***			4	104	221	68.0	56.2	1.3	14.0	89.9
12			5	93	106	53.2	51.7	1.5	14.1	65.3

Where n. s. means not soluble.

## Data Availability

Data are contained within the article.

## Supplementary Materials

The following supporting information can be downloaded at: https://www.mdpi.com/article/10.3390/ma16237319/s1. Figure S1: ^1^H NMR spectrum of the PGItc; Figure S2: ^13^C NMR spectrum of the PGItc in the range of carbon atoms of glycerol together with the ways of substitution of glycerol; Figure S3: ^13^C NMR spectrum of the PGItc in the range of carbonyl atoms; Figure S4: Fragments of ^1^H NMR spectra of the crosslinking products with 1,8-ODA (0.9–3.3 ppm) with the labeled protons of the used amine, taken in D_2_O; Figure S5: Fragments of ^1^H NMR spectra of the crosslinking products with 1,8-ODA (3.3–5.7 ppm), taken in D_2_O; Figure S6: Fragments of ^1^H NMR spectra of the crosslinking products with 1,8-ODA (5.3–7.7 ppm), taken in D_2_O; Figure S7: Formation of primary diamine salt; Table S1: Conditions and results of performed crosslinking processes for PGItc; Table S2: FTIR signals of the aza-Michael adduct, poly(glycerol itaconate) and 1,8-octanediamine; Table S3: Glass transition temperature during first and second heating of PGItc and crosslinked samples based on DSC analysis; Table S4: TG analysis DSC analysis of crosslinked samples.

## Abbreviations

A1: 1,2-ethylenediamine (1,2-EDA), A2: 1,4-butandeiamine (1,4-BDA), A3: 1,6-hexanediamine (1,6-HDA), A4: 1,8-octanediamine (1,8-ODA), A5: 1,10-decanediamine (1,10-DDA), Ac_Cit_: citraconic acid, Ac_Itc_: itaconic acid, Ac_Mes_: mesaconic acid, AN: acid number, ATR: Attenuated Total Reflectance, CPCs: calcium phosphate-based cements, DEA: diethylamine, DM_Cit_: dimethyl citraconate, DM_Itc_: dimethyl itaconate, DM_Mes_: dimethyl mesaconate, ED_tit_: esterification degree (by titration), ED_NMR_: esterification degree (by NMR analysis), EN: ester number, G: glycerol, %Is_Mes_: contribution of isomerization to a mesaconic compound, M1: aza-Michael monoadduct, M2: crosslinked aza-Michael product, %O_rd_: contribution of Ordelt reaction, P1: poly(glycerol maleate) (PGMal), P2: poly(glycerol fumarate) (PGF), P3: poly(glycerol itaconate) (PGItc), PMMA: poly(methyl methacrylate), PPM: post-polymerization modification, PTSA: p-toluenesulfonic acid, Zn(OAc)_2_: zinc acetate, %X_13C_^NMR^: degree of itaconic acid conversion.
