# Supplementary material for: Poly(glycerol itaconate) Crosslinking via the aza-Michael Reaction—A Preliminary Research
Source: Materials (Basel). 2023 Nov 24;16(23):7319. doi: 10.3390/ma16237319 (PMC10707276; doi:10.3390/ma16237319)
Supplement: Supplementary file 1 [file materials-16-07319-s001.zip › materials-2673862-supplementary.pdf]

Application of the aza-Michael reaction to crosslinking poly(glycerol itaconate) – a preliminary research

Magdalena Miętus[1], Krzysztof Kolankowski[1], Tomasz Gołofit[1], Paweł Ruśkowski[1], Marcin Mąkosa-Szczygieł[2], Agnieszka Gadomska-Gajadhur[1]*

[1]Faculty of Chemistry of Warsaw University of Technology; Noakowskiego 3 Street, 00-664 Warsaw, Poland; magdalena.mietus.dokt@pw.edu.pl (M.M.); krzysztof.kolankowski.dokt@pw.edu.pl (K.K.); tomasz.golofit@pw.edu.pl (T.G.); pawel.ruskowski@pw.edu.pl (P.R.)

[2]Faculty of Natural Sciences, Department of Chemistry, Norwegian University of Science and Technology, 7034 Trondheim, Norway; marcin.k.makosa-szczygiel@stud.ntnu.no

[*]Correspondence: agnieszka.gajadhur@pw.edu.pl

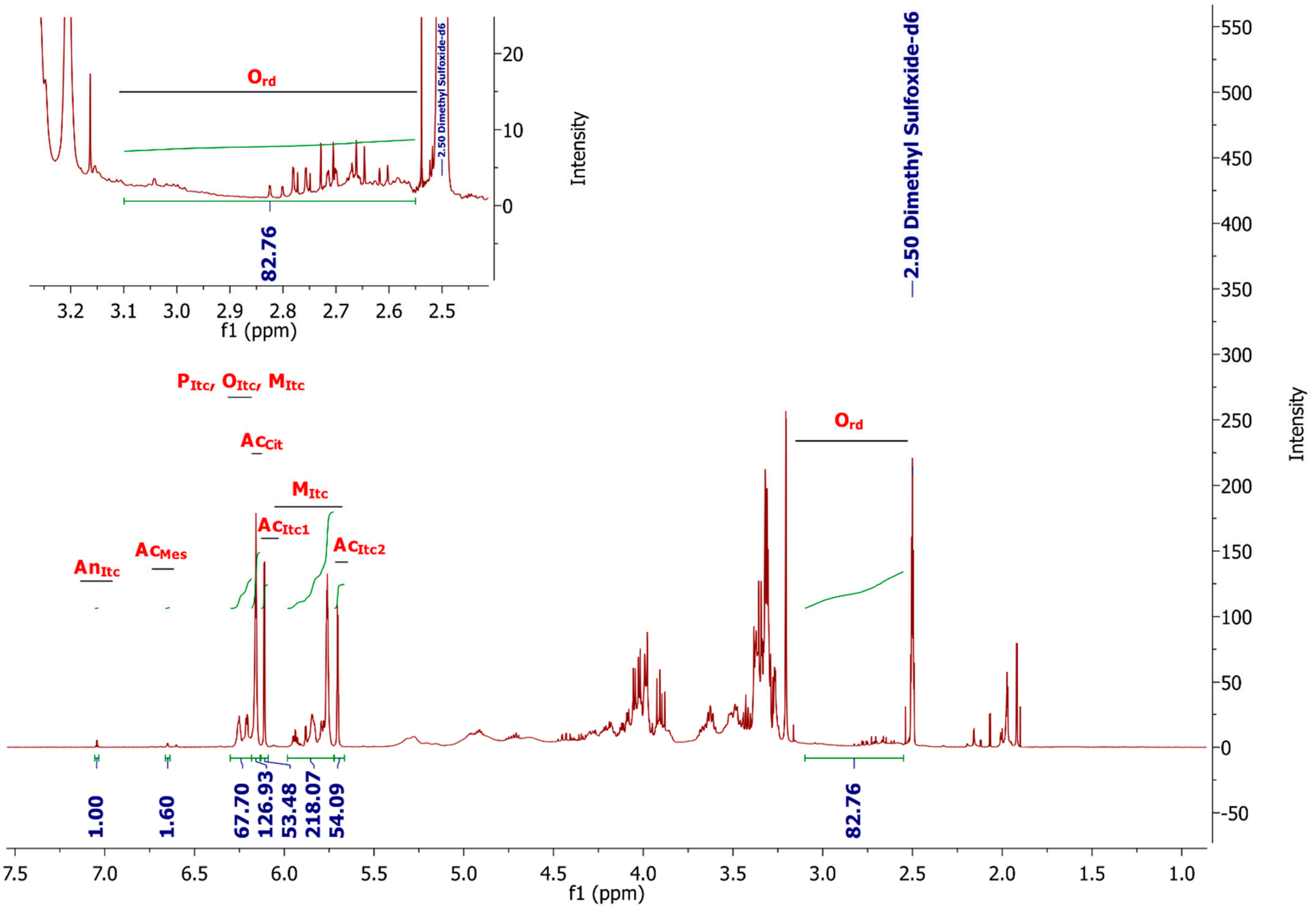

Figure S1. ^{1}H NMR spectrum of the PGItc.

To calculate the esterification degree with the use of NMR spectra the following equation was used:

$$ED_{NMR} = (\int P_{itc} + \int M_{itc}) / (\int P_{Itc} + \int M_{itc} + \int Ac_{Itc1} + \int Ac_{Itc2})) \times 100\% \quad (S1)$$

To calculate the proportion of isomerisation reactions of itaconic units to mesaconic units the following formula was used:

$$\%Is_{Mes} = (\int Ac_{Mes} / (\int Ac_{Mes} + ½ \times \int P_{Itc} + ½ \times \int M_{Itc})) \times 100\% \quad (S2)$$

The following equation was used to calculate the contribution of the Ordelt reaction:

$$\%O_{rd} = ((1/3 \times \int O_{rd}) / (1/3 \times \int O_{rd} + 1/2 \times \int P_{Itc} + 1/2 \times \int M_{Itc} + \int Ac_{Mes} + \int Ac_{Cit}) \times 100\% \qquad (S3)$$

Where:

$\int P_{Itc}$ – The value of the integral of the signal is from the itaconic polyesters, oligoesters, monoesters;

$\int M_{Itc}$ – The value of the integral of the signal is from the itaconic monoesters;

$\int Ac_{Itc1}$ – The value of the integral of the signal is from the itaconic acid;

$\int Ac_{Itc2}$ - The value of the integral of the signal is from the itaconic acid;

$\int An_{Itc}$ - The value of the integral of the signal is from the itaconic anhydride;

$\int Ac_{Mes}$ – The value of the integral of the signal is from the mesaconic acid;

$\int Ac_{Cit}$ - The value of the integral of the signal is from the citraconic acid;

$\int O_{rd}$ – The value of the integral of the signal is from the Ordelt protons.

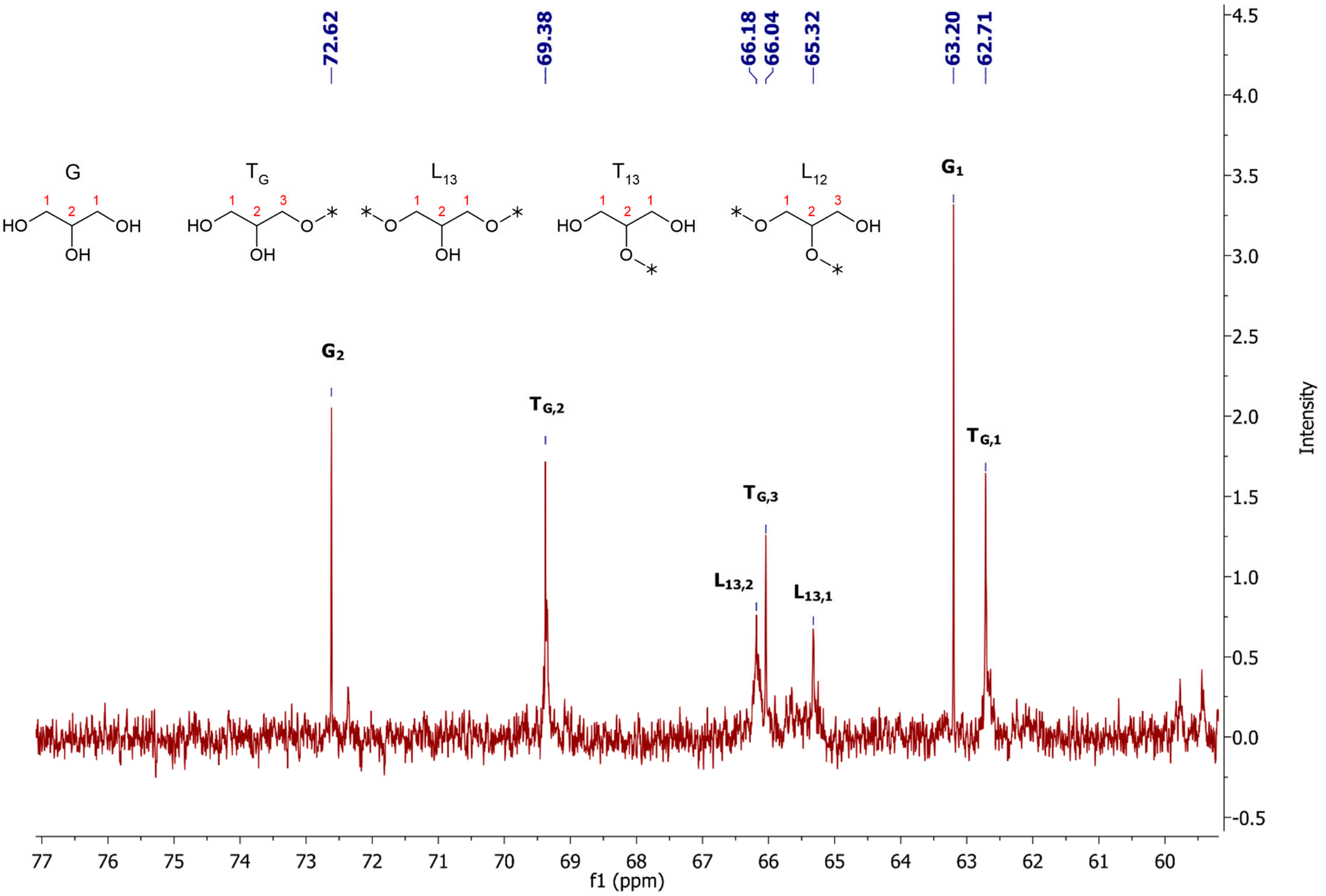

Figure S2. ^{13}C NMR spectrum of the PGItc in the range of carbon atoms of glycerol together with the ways of substitution of glycerol.

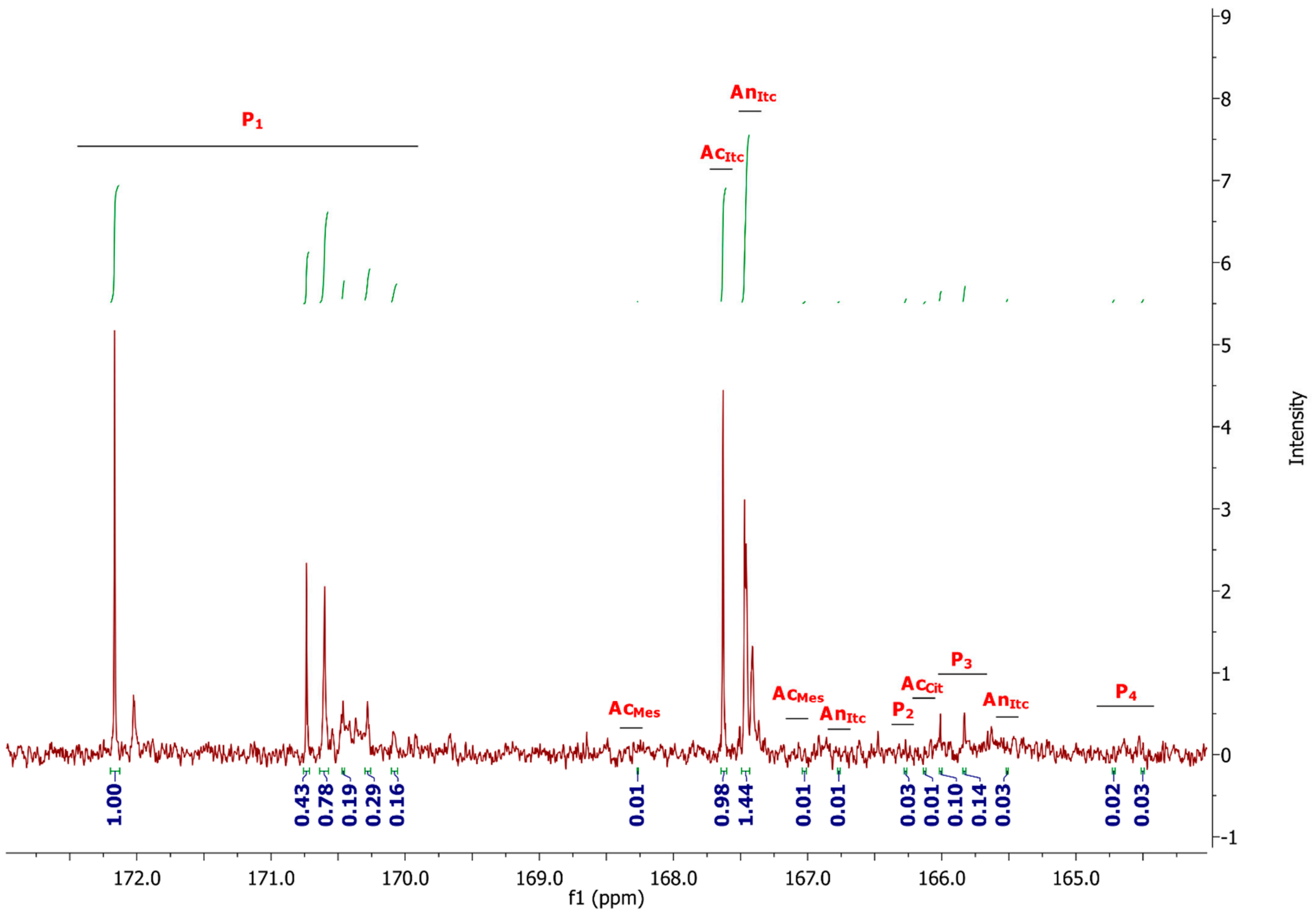

Figure S3. ^{13}C NMR spectrum of the PGItc in the range of carbonyl atoms.

The following equation was used to calculate the itaconic acid conversion degree:

$$\%X_{13C}^{NMR} = ((\int P_1 + \int P_2 + \int P_3 + \int P_4) / (\int P_1 + \int P_2 + \int P_3 + \int P_4 + \int Ac_{Itc})) \times 100\% \qquad (S4)$$

Where:

$\int P_1 + \int P_2 + \int P_3 + \int P_4$ – the value of the integrals of the signals from the itaconic polyesters, oligoesters and monoesters.

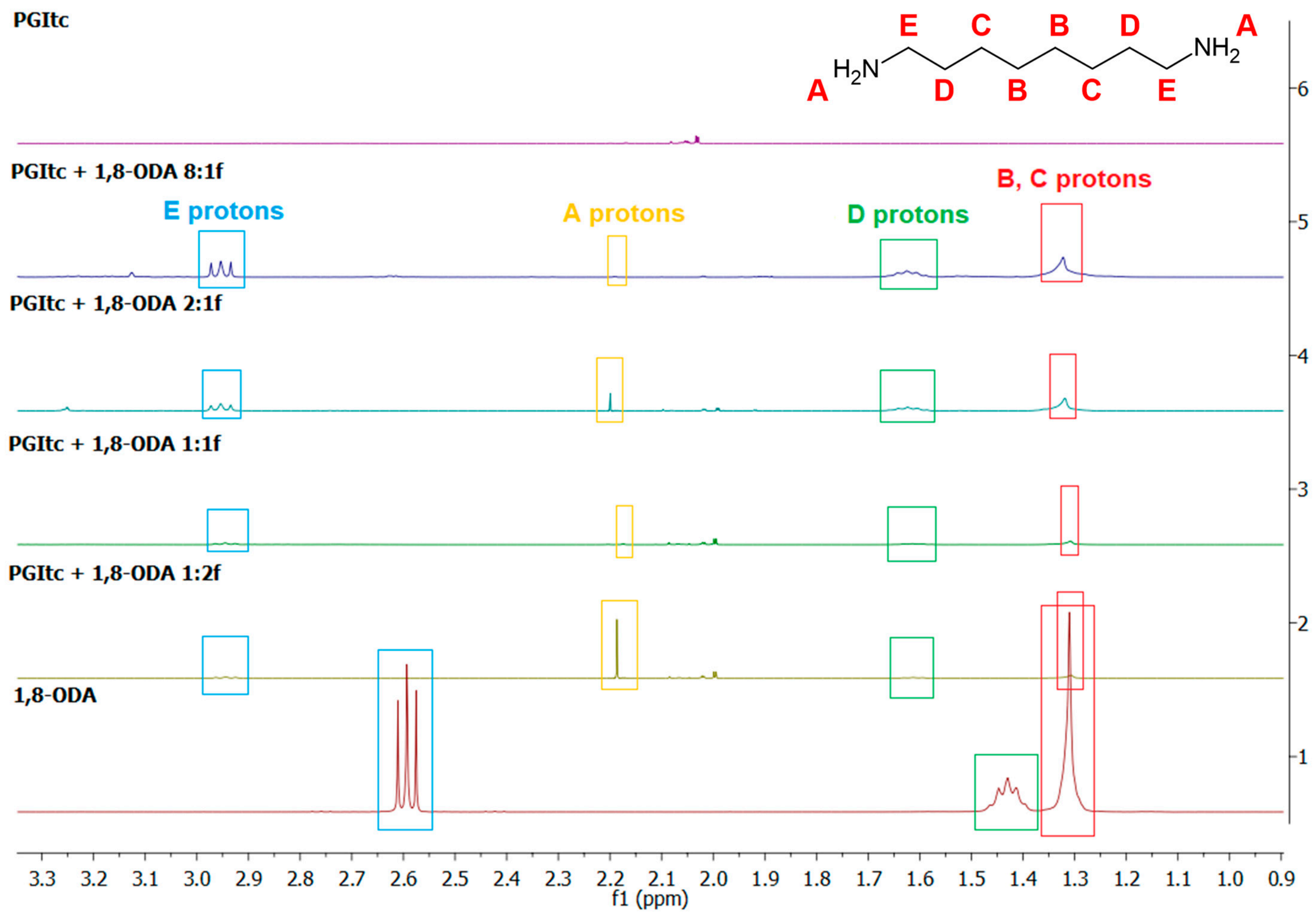

Figure S4. Fragments of ^{1}H NMR spectra of the crosslinking products with 1,8-ODA (0.9 - 3.3 ppm) with the labeled protons of the used amine, taken in D_2O.

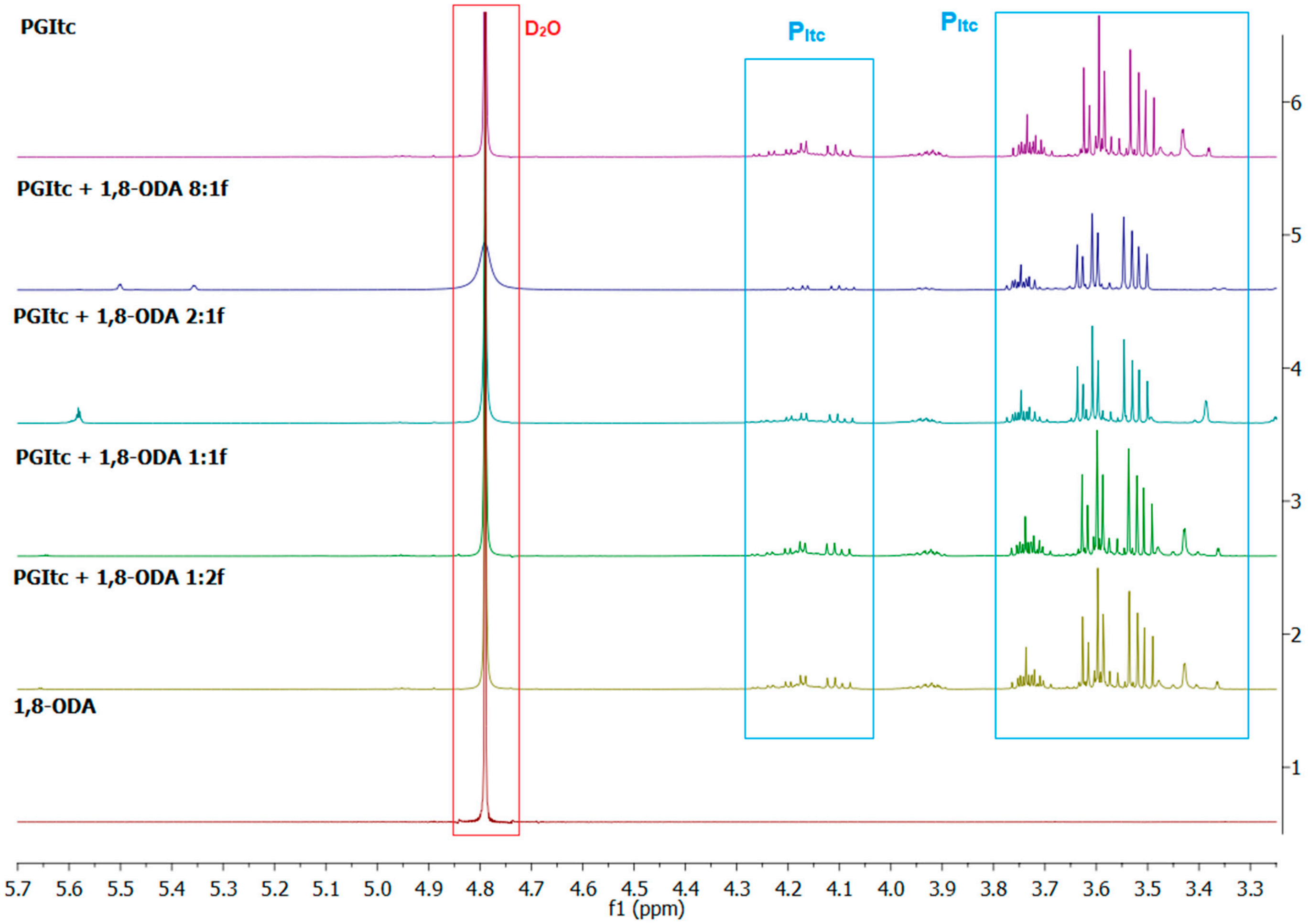

Figure S5. Fragments of ^{1}H NMR spectra of the crosslinking products with 1,8-ODA (3.3 – 5.7 ppm), taken in D_2O.

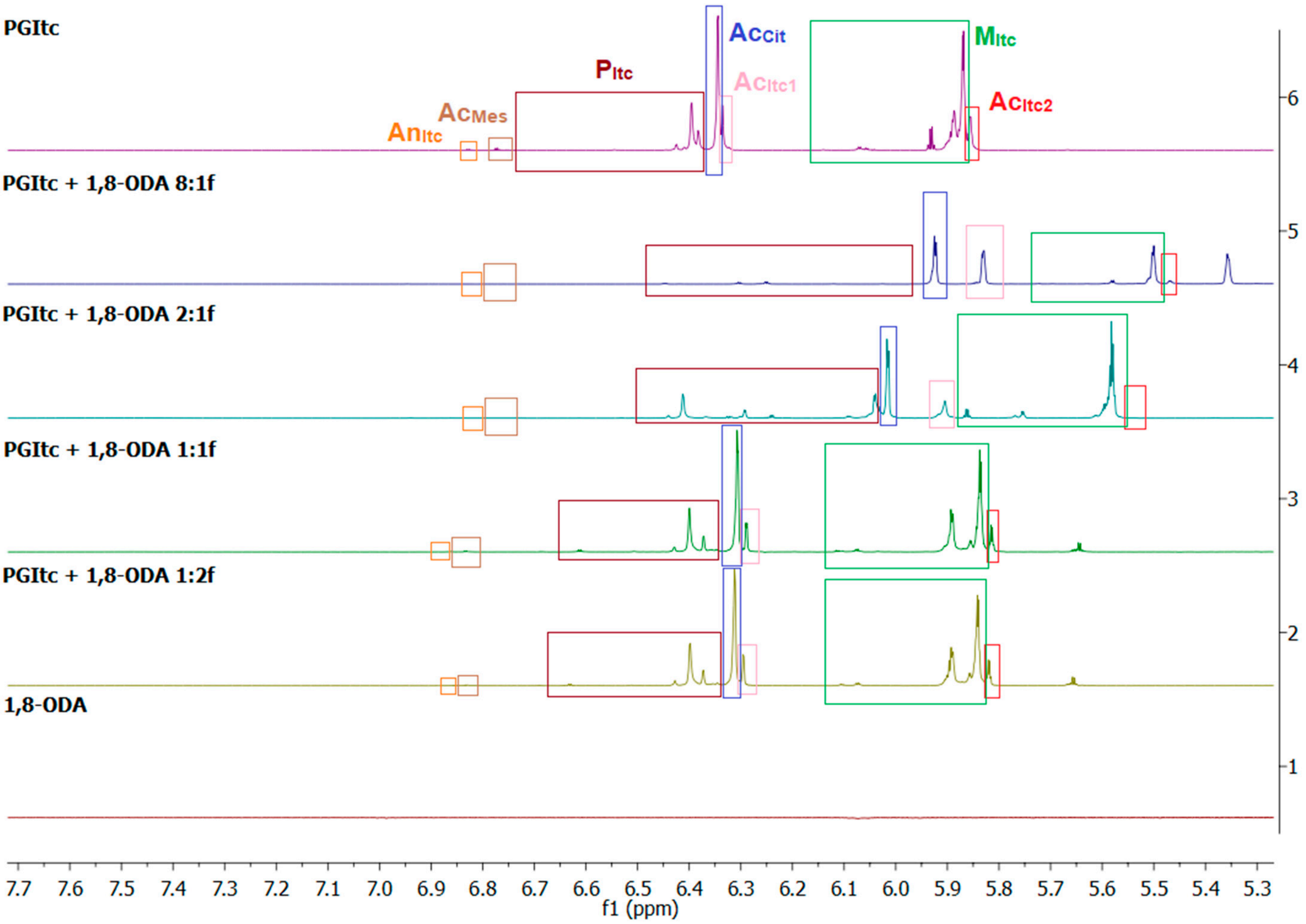

Figure S6. Fragments of ^{1}H NMR spectra of the crosslinking products with 1,8-ODA (5.3 – 7.7 ppm), taken in D_2O.

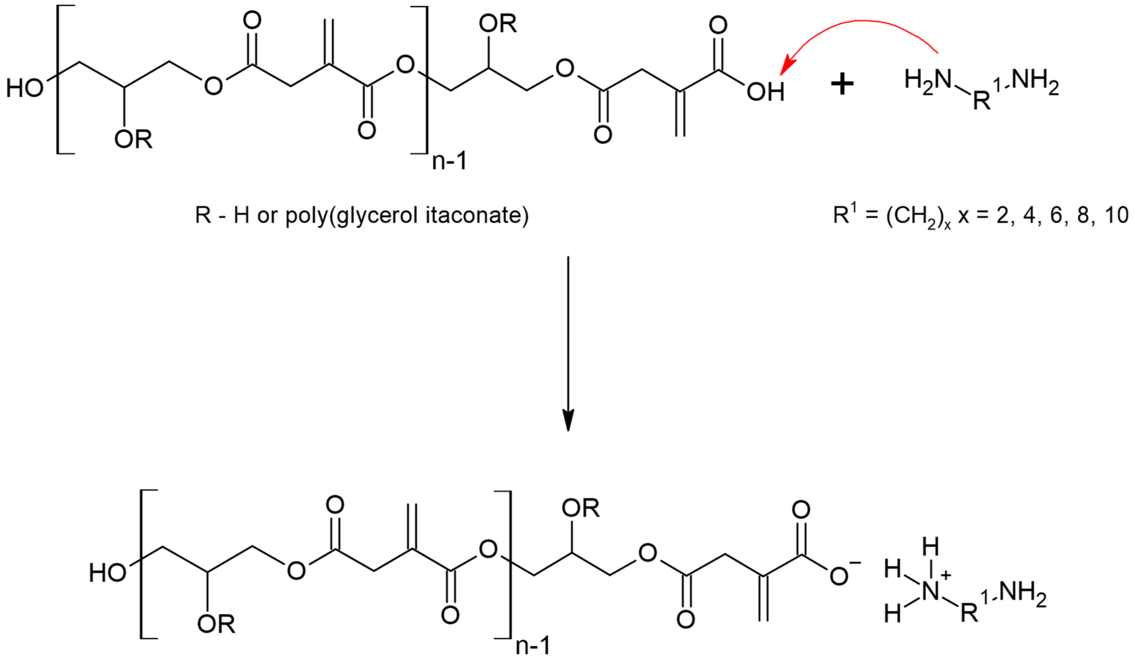

Figure S7. Formation of primary diamine salt.

The calculations performed to determine the required amount of *t*-BuOH ($m_{t\text{-BuOH}}$):

The required $m_{t\text{-BuOH}}$ was calculated based on the determined AN value for the PGItc sample (AN = 135.6). It corresponded to the number of milligrams of KOH that was required to esterify the free acid groups presented in 1 g of the test sample. To determine $m_{t\text{-BuOH}}$, the number of moles of KOH (n_{KOH}) per reactor charge was determined (m_w = 35 g):

$$n_{KOH} = ((AN_{titr} / 1000) / 56.1) \cdot m_w \quad (S5)$$

The required amount of *t*-BuOH was determined, taking into account the 15% excess:

$$m_{t\text{-BuOH}} = (n_{KOH} + 0.15 \cdot n_{KOH}) \cdot 74.1 \quad (S6)$$

Table S1. Conditions and results of performed crosslinking processes for PGItc.

Functionality (Signature)	Amine	Maximum crosslinking temperature [°C]	Time of crosslinking [s]	Visual evaluation of product viscosity
1:2f (a1)	A1	55	49	+
1:1f (a2)		57	54	++
2:1f (a3)		68	54	+
8:1f (a4)		65	84	+
1:2f (b1)	A2	41	54	+++
1:1f (b2)		55	73	+
2:1f (b3)		60	120	+++
8:1f (b4)		65	60	+
1:2f (c1)	A3	40	56	++
1:1f (c2)		41	89	++
2:1f (c3)		46	118	++
8:1f (c4)		62	185	+
1:2f (d1)	A4	34	46	+++
1:1f (d2)		35	37	+++
2:1f (d3)		50	41	+++
8:1f (d4)		49	45	++
1:2f (e1)	A5	38	95*	+
1:1f (e2)		42	60*	+
2:1f (e3)		50	50*	+
8:1f (e4)		82	40*	+

Where

* The amine started to crystallise during mixing with the polymer sample

+++ Product is well dense, does not stretch without the use of force

++ Product is partially dense, weakly stretches without the use of force

+ Product is easily extractable without the use of force

Table S2. FTIR signals of the aza-Michael adduct, poly(glycerol itaconate) and 1,8-octanediamine.

Wavenumber ν [cm^{-1}]	Observed vibration	Symbol
3200–3550	O-H bond stretching vibrations	A and A'
3300–3500	N-H bond stretching vibrations	B
2960–2925	C-H bond stretching vibrations of the methylene group	C, C' and C''
1730-1715	Stretching vibrations of the C=O carbonyl group of α,β-unsaturated esters	D

	Stretching vibrations of the C=O carbonyl group of esters	D’
1680-1630	Stretching vibrations of the C=C double bond	E and E’
~1560	N-H bending vibration of NH_3^+ group	F
~1382	Deformation vibration of the C-H bond of the methylene group	G and G’
~1156	C-O bond stretching vibrations of the acyl group	H and H’
~1035	C-O bond stretching vibrations of the alkoxy group	I and I’

Table S3. Glass transition temperature during first and second heating of PGItc and crosslinked samples based on DSC analysis.

**Amine**	T_{g1} **[°C]**	T_{g2} **[°C]**	ΔT_g ($T_{g1} - T_{g2}$) **[°C]**
-	-58.0	-42.3	-15.7
A2	-48.6	-48.5	-0.1
A3	-53.7	-44.0	-9.7
A4	-51.8	-44.0	-7.8
A5	-51.4	-46.6	-4.8

Table S4. TG analysis DSC analysis of crosslinked samples.

**Amine**	T_d **5% [°C]**	T_d **50% [°C]**	T_d **85% [°C]**
A2	144.5	283.9	469.5
A3	141.2	262.3	457.4
A4	130.6	276.5	422.0
A5	141.7	267.8	452.8
